# Time Lag Between Light and Heat Diurnal Cycles Modulates *CIRCADIAN CLOCK ASSOCIATION 1* Rhythm and Growth in *Arabidopsis thaliana*

**DOI:** 10.3389/fpls.2020.614360

**Published:** 2021-02-11

**Authors:** Kosaku Masuda, Tatsuya Yamada, Yuya Kagawa, Hirokazu Fukuda

**Affiliations:** ^1^Graduate School of Engineering, Osaka Prefecture University, Osaka, Japan; ^2^Japan Society for the Promotion of Science, Tokyo, Japan

**Keywords:** circadian clock, dark cycle, entrainment, phase response curve, singularity response, synchronization

## Abstract

Plant growth responses to cues such as light, temperature, and humidity enable the entrainment of the circadian rhythms with diurnal cycles. For example, the temperature variations between day and night affect plant growth and accompany the time lag to light cycle. Despite its importance, there has been no systematic investigation into time lags, and the mechanisms behind the entrainment of the circadian rhythms with multiple cycles remain unknown. Here, we investigated systemically the effects of the time lag on the circadian rhythm and growth in *Arabidopsis thaliana*. To investigate the entrainment status of the circadian clock, the rhythm of the clock gene *CIRCADIAN CLOCK ASSOCIATION 1* (*CCA1*) was measured with a luciferase reporter assay. As a result, the rhythm was significantly modulated by the time lag with +10°C heating for 4 h every day but not −10°C cooling. A model based on coupled cellular oscillators successfully described these rhythm modulations. In addition, seedling growth depended on the time lag of the heating cycle but not that of the cooling cycle. Based on the relationship between the *CCA1* rhythms and growth, we established an estimation method for the effects of the time lag. Our results found that plant growth relates to the *CCA1* rhythm and provides a method by which to estimate the appropriate combination of light–dark and temperature cycles.

## Introduction

Organisms on earth have been found to have circadian clocks that are adapted to 24 h periods for environmental cycles. This includes plants, whose circadian clocks have important roles in various physiological processes, such as photosynthesis and flowering (Dodd et al., 2014; Creux and Harmer, 2019). The plant circadian clock responds to environmental changes and is entrained by the diurnal light–dark cycle (Webb et al., 2019). Entrainment to the light–dark cycle provides some advantages to plants, namely, circadian resonance, in which plants grow larger in an environment with periods that are similar to the intrinsic period of the circadian clock (Dodd et al., 2005; Higashi et al., 2015; Resco de Dios et al., 2016).

In nature and horticultural practices, the light–dark cycle is usually accompanied by a temperature cycle, which is associated with a day temperature that is higher than the night temperature. The temperature cycle is another strong entrainment cue (zeitgeber) for the plant circadian clock. In horticultural studies, the phase differences between light and temperature cycles have been found to have crucial effects on plant growth. Most plants exhibit optimal growth with the in-phase regime, in which the day temperature is higher than the night temperature (which we have termed +DIF) (Xiong et al., 2011, Xiao et al., 2018). The antiphase regime, that is, when the night temperature is higher than the day temperature (which we have termed −DIF), suppresses the elongation growth of the stems and the leaves of various plant species (Thingnaes et al., 2003; Xiong et al., 2011). Therefore, +DIF conditions are preferable for plant growth. However, time lags between light and temperature cycles can frequently occur due to unsettled weather. Furthermore, in horticultural facilities, the time lag is also artificially generated by supplemental lighting at night or cooling during the day (Anpo et al., 2018; Kozai et al., 2019). The spatial unevenness under lighting or air conditioning and the thermal conductivity of air and soil/water might also generate a spatial time lag in DIF. As such time lags might affect plant production yields, the elucidation of their effects is required to improve horticultural practices. However, there has been no systematic investigation into the time lag of temperature cycles and the entrainment behavior of circadian rhythms under dual-zeitgeber cycles.

Variations in the circadian rhythm due to environmental stimuli have been addressed using the phase response curve (PRC) (Johnson et al., 2003). The PRC describes the extent of phase shift as a function of the phase with a stimulus. This provides significant information for entrainment, for example, the entrainment range and stable locking phase for environmental cycles (Johnson et al., 2003; Granada et al., 2009). In previous studies, PRCs for darkness and temperature stimuli have been reported (Michael et al., 2003; Fukuda et al., 2008; Thines and Harmon, 2010). If the application duration of the stimulus is sufficiently short (e.g., heating for 4 h), the effect of the stimulus will be relatively weak on the circadian rhythm (Masuda et al., 2021). Consequently, the rhythm can be modulated to various forms by using weak stimuli. This modulation can be estimated numerically using a phase oscillator model (Fukuda et al., 2013). In contrast, if the duration of the applied stimulus is long, the effects are too strong to modulate the circadian rhythm into various forms. Therefore, to elucidate the rhythm modulation using dual zeitgebers, it is appropriate that the duration of the temperature stimuli be short.

In this study, the promoter activity rhythms of *CIRCADIAN CLOCK ASSOCIATED1* (*CCA1*) were measured to investigate the effects of time lags on the circadian rhythm. *CCA1* is one of the core clock genes of the plant circadian clock and is closely related to stress responses (Lai et al., 2012). In addition, the PRC of the *CCA1* rhythms for various stimuli have been measured in previous studies (Fukuda et al., 2008; Ohara et al., 2015; Masuda et al., 2017, 2021). We analyzed the *CCA1* rhythm in *Arabidopsis thaliana* under long-day conditions (L/D = 16/8 h) with periodic temperature stimuli (±10°C for 4 h). To elucidate the modulation of the *CCA1* rhythms using this time lag, we simulated numerically the rhythm using a phase oscillator model. In addition, we also measured the fresh weight of the seedlings and their leaf area to evaluate the effects of time lag on seedling growth.

## Materials and Methods

### Measurement of *CCA1* Rhythms

**FIGURE 1:**
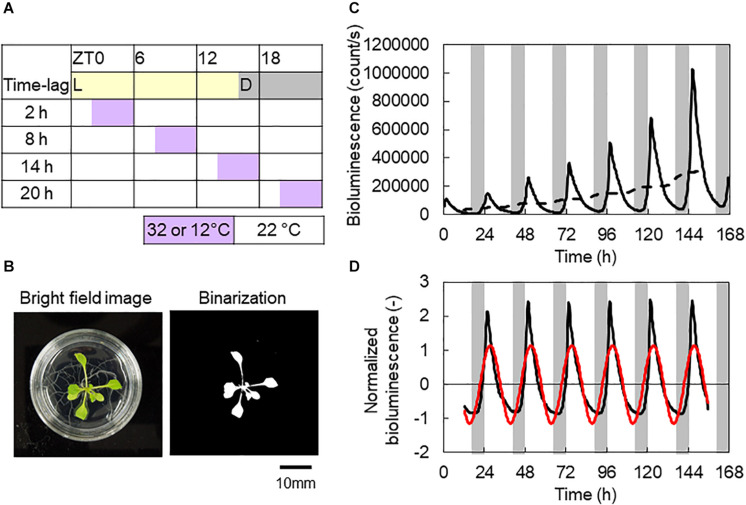
Experimental conditions and representative analysis data. **(A)** Time table of L/D = 16/8 h and 4 h ± 10°C conditions. **(B)** Bright-field and binarization images of an *Arabidopsis thaliana* seedling. Projected leaf area (PLA) measured by banalization of the areal part. **(C)** Bioluminescence of a *CCA1::LUC* seedling. Solid and broken lines are raw data and its moving average, respectively. **(D)** Normalized bioluminescence (black line) and the first Fourier series (red line). Data were from control conditions with in-phase initial conditions **(B–D)**.

To investigate the *CCA1* rhythms in each individual, we utilized transgenic *A. thaliana CCA1*::*LUC*, which carries luciferase reporters driven by the promoters of the clock gene *CCA1* (Nakamichi et al., 2004). Plants were grown on gellan gum-solidified Murashige–Skoog medium (MS Plant Salt Mixture, Wako Chemical Co.) at the standard concentration with 2% (w/v) sucrose in 40-mm-diameter dishes (one individual per dish) under L/D = 12/12 h and 100 μmol m^–2^ s^–1^ fluorescent white light at 22 ± 0.5°C for 7 days. The plants were treated with 500 μl of 1 mM luciferin (D-Luciferin Firefly, potassium salt, BIOSYNTH AG, in water) 24 h before the start of bioluminescence monitoring. Bioluminescence measurements were carried out using an automatic luminescence measuring system known as *Kondotron* with 20 plants under light-emitting diode (LED) illumination with a red LED (60 μmol m^–2^ s^–1^ light conditions, λ_p_ = 660 nm) and blue LED (15 μmol m^–2^ s^–1^ under light conditions, λ_p_ = 470 nm) at 22 ± 0.5°C for 7 days (Kondo et al., 1993; Fukuda et al., 2008). The photoperiod was set to L/D = 16/8 h, and the first dark period was started 16 h after the beginning of the measurements as an in-phase initial condition. Periodic temperature stimuli (+10 or −10°C from 22°C for 4 h within a 24 h period) were applied with four time lags (Δ*t* = 2, 8, 14, and 20 h after turning on the light, referred to as “light on”), as shown in Figure 1A. In addition, as an antiphase initial condition, the first dark period was started 4 h after the measurements began, where the *CCA1* rhythm started with an inversed phase to the light–dark cycle. As controls, experiments were also performed using L/D = 16/8 h conditions under both in-phase and antiphase initial conditions at 22°C without temperature cycles. The heating and the control of the in-phase initial conditions were carried out once. The cooling and the control of the antiphase initial conditions were repeated two times. After measuring the bioluminescence, we measured plant size using both fresh weight of the areal part and projected leaf area (PLA) (Figure 1B), to evaluate the effects on productivity for commercial agriculture (Kozai et al., 2019).

### Oscillation Analysis

Bioluminescence of the *CCA1* rhythm was initially normalized as follows:

(1) $${\bar{l}}_{i}=\frac{1}{2\,w+1}\,\sum_{k=-w}^{w}l_{i+k},$$

(2) $$L_{i}=\left(l_{i}-{\bar{l}}_{i}\right)/{\bar{l}}_{i}$$

where *l*_i_ is the *i*th time point of bioluminescence and ${\bar{l}}_{\text{i}}$ is the moving averaged bioluminescence (Figure 1C). *w* is the half window size of the moving average. The measurement intervals were 20 min, so *w* was set to 36 for the 24 h window averaging. *L*_i_ is the normalized bioluminescence (Figure 1D).

Then, to determine the amplitude and phase of the *CCA1* rhythm, we obtained the first Fourier series, *A* cos *θ*(*t*), of *L*_i_ (red cosine curve in Figure 1D). The first Fourier component was obtained with the following equations:

$$a_{1}=\frac{2}{h-2\,w}\,\sum_{i=1+w}^{h-w}L_{i}\,\cos\left(2\,\pi \,\frac{i\,\Delta \,s}{T}\right),$$

(3) $$b_{1}=\frac{2}{h-2\,w}\,\sum_{i=1+w}^{h-w}L_{i}\,\sin\left(2\,\pi \,\frac{i\,\Delta \,s}{T}\right),$$

where *T* = 24 h and the measurement interval Δ*s* = 1/3 h. *h* is the number of the time course data. Using *a*_1_ and *b*_1_, the amplitude *A* and phase *θ*(*t*) were determined as follows:

(4) $$A=\sqrt{a_{1}^{2}+b_{1}^{2}},$$

(5) $$\theta \,\left(t\right)=2\,\pi \,\frac{t}{T}-\theta_{1},$$

(6) $$\theta_{1}=\tan^{-1}\frac{b_{1}}{a_{1}}.$$

where *θ*_1_ indicates the phase delay in the *CCA1* rhythm in response to the light–dark cycles, that is, the locking phase, which appeared at light-on [*θ*(0) = −*θ_*1*_*]. We used the Tukey–Kramer test for multiple comparisons for *A* at a significance level of 0.05. We also used the Watson–Williams test with Bonferroni correction for multiple comparisons for *θ*_1_ at a significance level of 0.05 (Watson and Williams, 1956). The mean of locking phase ${\bar{\theta }}_{1}$ (which is termed the circular mean) is defined as $\arg\left\{\frac{1}{N}\,\sum_{j=1}^{N}e^{\text{i}\,\theta_{1,j}}\right\}$, where *θ*_1,_*_j_* is the locking phase (rad) of the *j*th individual.

### Numerical Simulation of Circadian Rhythm Modulations

Since the plant circadian clock has an enormous number of cellular oscillators, the individual-level circadian rhythms represent synchronization among the oscillators (Fukuda et al., 2013). In addition, for the dual-zeitgeber cycles, each oscillator is modulated through the multiple phase responses. Therefore, the population dynamics of the cellular oscillators and their synchronization states are described as follows:

(7) $$\frac{d\,\phi_{j}}{d\,t}=\omega_{j}+p_{\text{D}}\,\left(t\right)\,Z_{\text{D}}\,\left(\phi_{j}\right)+p_{\text{T}}\,\left(t\right)\,Z_{\text{T}}\,\left(\phi_{j}\right)+\frac{K}{N}\,\sum_{k=1}^{N}\text{sin}\,\left(\phi_{k}-\phi_{j}\right),$$

(8) $$R\,\left(t\right)\,e^{\text{iΦ}\,\left(t\right)}=\frac{1}{N}\,\sum_{j=1}^{N}e^{\text{i}\,\phi_{j}\,\left(t\right)},$$

where *ϕ_j_* and *ω_j_* are the phase and natural frequency of the *j*th oscillator, respectively. *K* represents the coupling strength, and *N* is the number of oscillators. *ω_j_* takes a normal distribution with a standard deviation σ_ω_ and a mean value *ω*_0_. *p*_D_(*t*) and *p*_T_(*t*) indicate the presence of stimulus for 8 h dark and 4 h +10°C or 4 h −10°C stimuli, respectively [*p*(*t*) = 1 for stimulus on and *p*(*t*) = 0 for stimulus off]. They are described as follows:

(9) $$p_{\text{D}}\,\left(t\right)=\left\{ \begin{array}{c} 1,16+24\,m\leq t<24+24\,m\\ 0,o\,t\,h\,e\,r \end{array}\right.$$

(10) $$p_{\text{T}}\,\left(t\right)=\left\{ \begin{array}{c} 1,\Delta \,t+24\,m\leq t<\Delta \,t+4+24\,m\\ 0,o\,t\,h\,e\,r \end{array}\right.$$

where *m* is an integer. *Z*_D_(*ϕ*) and *Z*_T_(*ϕ*) are the phase sensitivity functions for dark and temperature stimuli, respectively (Masuda et al., 2021). They are described using the same formula, as follows: *Z*(*ϕ*) = *a* sin(*ϕ* − *α*). We used the previously obtained values of *a* and *α* for 8 h darkness and 4 h ± 10°C stimuli (Masuda et al., 2021). In addition, the collective rhythm *X* of the oscillators is denoted as $X\,\left(t\right)=\frac{1}{N}\,\sum_{j=1}^{N}\cos\left(\phi_{j}\,\left(t\right)\right)=R\,\left(t\right)\,\cos\left(\text{Φ}\,\left(t\right)\right)$, where R(*t*) and Φ(*t*) correspond to the amplitude and phase of the individual-level rhythms, respectively. In this study, parameters were set as *N* = 1,000, *K* = 0.01, *ω*_0_ = 2π/23 h^–1^ rad, and σ_ω_ = 0.2*ω*_0_.

## Results

### Modulations of the *CCA1* Rhythms by the Temperature Cycles

**FIGURE 2:**
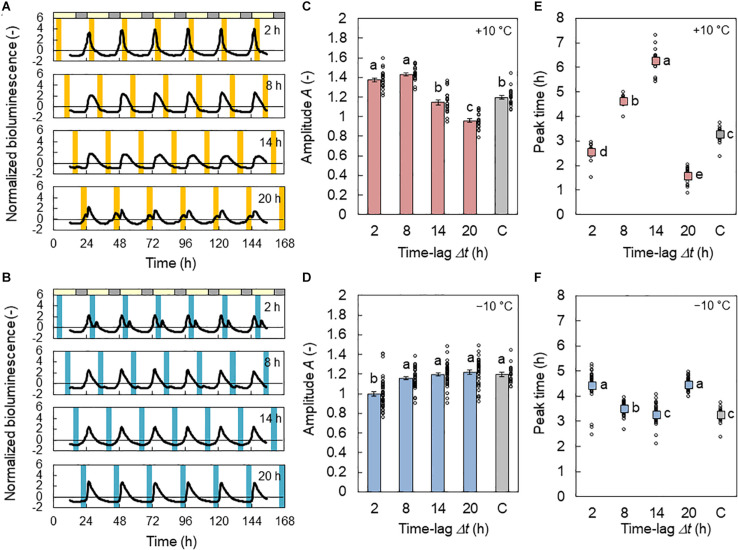
Effect of time lag on the *CCA1* rhythm in conditions of L/D = 16/8 h and 4 h ± 10°C cycles for the in-phase initial conditions. **(A,B)** Normalized bioluminescence of *CCA1*::*LUC* in +10°C **(A)** and −10°C **(B)** conditions. **(C,D)** Amplitude *A* of the bioluminescence oscillation in +10°C **(C)** and −10°C **(D)** conditions (mean ± SEM, *n* = 20 individuals in +10°C, 40 in −10°C, and 20 in the control condition). The control condition is labeled C. The circles indicate the individual data points. Different letters indicate significant differences for each panel (Tukey–Kramer test, *p* < 0.05). **(E,F)** Peak time in +10°C **(E)** and −10°C **(F)** conditions (circular mean, *n* = 20 individuals in +10°C, 40 in −10°C, and 20 in the control condition). The circles indicate the individual data. Different letters indicate significant differences for each panel (Watson–Williams test with Bonferroni correction, *p* < 0.05).

In Figure 2A, the normalized bioluminescence of *CCA1*::*LUC* under L/D = 16/8 h with 4 h +10°C cycles is shown. At Δ*t* = 8 h (+10°C in days), the rhythm was amplified (Figure 2C) from the basic amplitude, which was 1.20 when the temperature cycle was absent. In contrast, at Δ*t* = 20 h (+10°C in late night), the *CCA1* rhythm was disturbed, and its amplitude was reduced. Figure 2B shows the *CCA1* rhythm under the cooling conditions (L/D = 16/8 h with 4 h −10°C cycles). Only at Δ*t* = 2 h (−10°C in early days) was the *CCA1* rhythm disturbed and reduced (Figure 2D). When the amount of amplitude change is compared (the maximum value minus minimum value), the effect of the −10°C condition was 53% smaller than that of the +10°C condition.

The peak times for the *CCA1* rhythms are shown in Figures 2E,F. For the +10°C condition, at Δ*t* = 2, 8, and 14 h, the peak appeared 2.5, 4.6, and 6.2 h after light on, respectively. Thus, the increment of Δ*t* increased the phase delay of the *CCA1* rhythm. In contrast, at Δ*t* = 20 h, the peak appeared after 1.6 h; that is, the phase delay substantially decreased. For the −10°C condition, at Δ*t* = 2, 8, and 14 h, the peak appeared at 4.4, 3.5, and 3.3 h after light on, respectively. Thus, the increment of Δ*t* provides a slight phase advance for the *CCA1* rhythm. However, at Δ*t* = 20 h, the peak appeared at 4.4 h; that is, the phase delay was increased. The amount of phase change between the maximum and minimum values of the peak times at −10°C was also 75% smaller than that at +10°C. Under the antiphase initial conditions, the changes in *A* and peak time were similar to those under the in-phase initial conditions (Supplementary Figure S1), but a small effect of initial conditions was observed due to a transient in the first LD cycle.

### Numerical Simulation of the Amplitude and Phase Modulations

**FIGURE 3:**
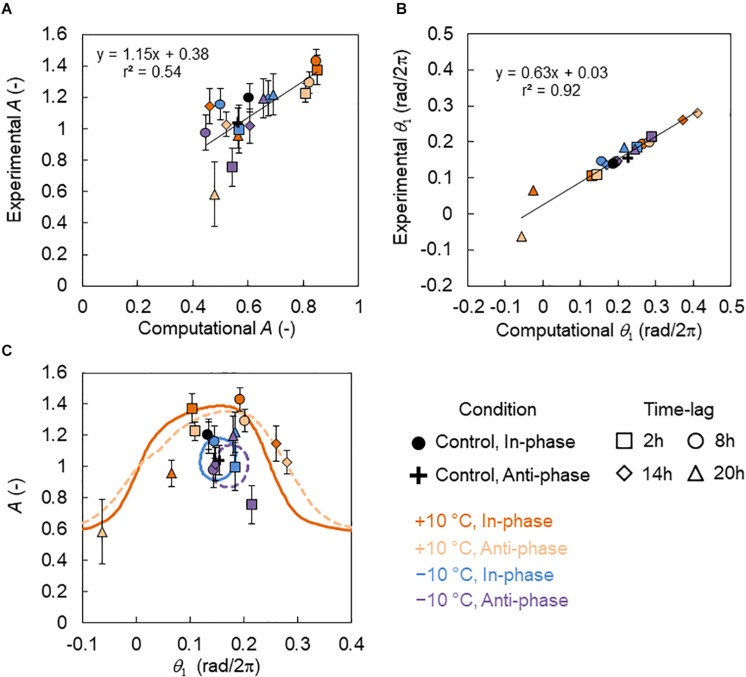
Relationship between the amplitude and locking phase of the *CCA1* rhythms in the experiments and simulations. **(A)** Correlation between amplitudes *A* in the experiment and simulation (mean ± SD in experiment). **(B)** Correlation between the locking phase *θ*_1_ in the experiment and the simulation (circular mean in experiment). The black lines indicate regression lines. **(C)** Relationship between *A* (mean ± SD) and *θ*_1_ (circular mean). The symbols indicate the experimental data points. The colored lines indicate the relationship between *A* and *θ*_1_ in the simulation. The solid lines indicate the in-phase conditions and the broken lines indicate the antiphase conditions. The simulation values of *A* and *θ*_1_ were calibrated using the regression lines in **(A)** and **(B)** as *A*_*calibrated*_ = 1.15*A* + 0.38 and *θ*_1,_*_*calibrated*_* = 0.63*θ*_1_ + 0.03.

We performed the numerical simulation using a phase oscillator model with PRCs (Eq. 7). Figure 3A shows the relationship between experimental and computational amplitudes *A*, and there was a high correlation (correlation coefficient *r* = 0.74, *p* < 0.01). However, experimental *A* was dispersed in the low-amplitude state (*A* < 0.6), indicating that amplitude estimation was difficult. Figure 3B, however, shows the relationship between the experimental and computational locking phase *θ*_1_, and there was a very high correlation (*r* = 0.96, *p* < 0.01).

The relationship between *A* and *θ*_1_ in the experiment and simulation is shown in Figure 3C, where the simulation values of *A* and *θ*_1_ were calibrated using the regression lines in Figures 3A,B. Under the +10°C condition, the value of *A* showed a peak around *θ*_1_ = 0.15 rad/2π, while under the −10°C conditions, *A* varied on a small loop. This indicates that the modification range at −10°C was smaller than that at +10°C.

### Effect of Time Lag on Growth

**FIGURE 4:**
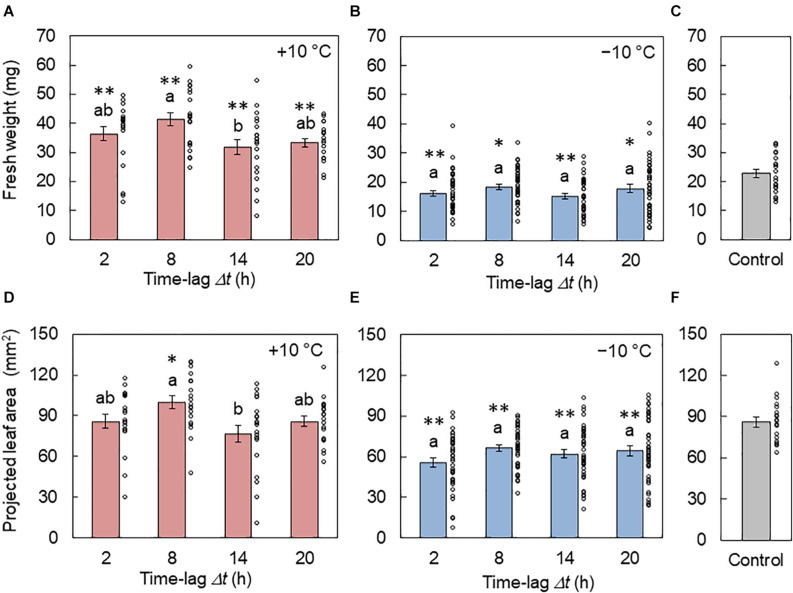
Effect of the time lag on plant growth in conditions of L/D= 16/8 h and 4 h ± 10°C cycles. **(A–C)** Fresh weights of the areal part from the +10°C **(A)**, −10°C **(B)**, and control **(C)** conditions (mean ± SEM, *n* = 20 individuals in **(A)** and **(C)** and 40 in **(B)**). **(D–F)** Projected leaf areas from the +10°C **(D)**, −10°C **(E)**, and control **(F)** conditions (mean ± SEM, *n* = 20 individuals in **(D)** and **(F)** and 40 in **(E)**. The circles indicate the individual data points. Two conditions that do not have the same letter indicate significant differences for each panel (Tukey–Kramer test, *p* < 0.05). Asterisks indicate significant differences with the control condition (Welch’s *t*-test, *p < 0.05, **p < 0.01).

In Figures 4A,B, the fresh weights of the areal part of plants from the +10 and −10°C conditions are shown, respectively. The weight was larger at Δ*t* = 8 h and smaller at *Δt* = 14 h under the +10°C condition. In contrast, there was no significant difference between the time lags under the −10°C condition. Figures 4D,E show the PLA under the +10 and −10°C conditions, respectively. Similar results were observed between the fresh weight and PLA. By the comparison of the control conditions, it was found that the +10°C cycle tended to enhance growth while the −10°C cycle suppressed growth.

**FIGURE 5:**
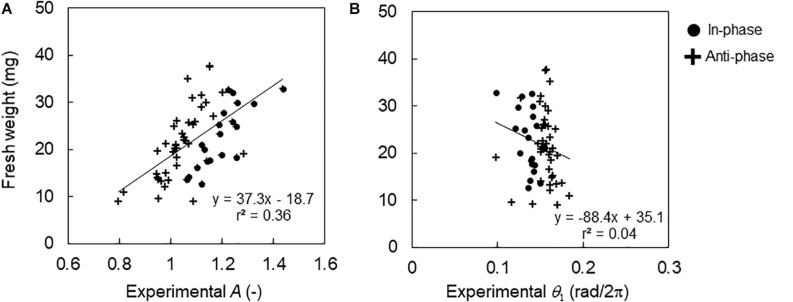
Relationship between fresh weight and *CCA1* rhythms in the control conditions. **(A)** Relationship between fresh weight and amplitude *A* in individuals. **(B)** Relationship between fresh weight and locking phase *θ*_1_ in the individuals. The black lines indicate regression lines.

Figure 5 shows the relationship between the fresh weight and the *CCA1* rhythm under the control conditions. The fresh weight increased with the increasing amplitude of *A* with a correlation (*r* = 0.60, *p* < 0.01). In contrast, the locking phase *θ*_1_ showed almost the same value; the mean values of phase ${\bar{\theta }}_{1}$ were 0.14 and 0.16 rad/2π (peak time 3.3 and 3.7 h) for the in-phase and antiphase initial conditions, respectively. Therefore, the fresh weight showed no correlation for *θ*_1_ (*r* = −0.20, *p* > 0.05).

**FIGURE 6:**
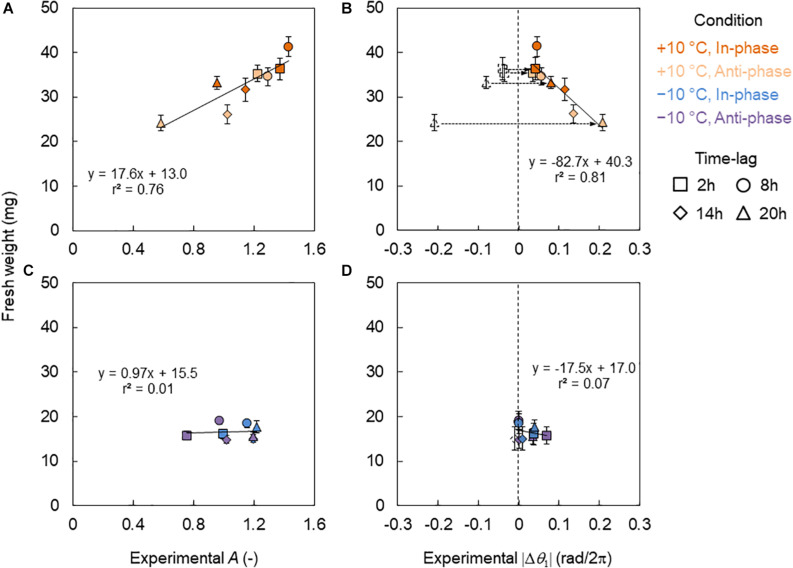
Relationship between fresh weights and *CCA1* rhythms in the conditions of L/D = 16/8 h and 4 h ± 10°C cycles. **(A,B)** Relationship between fresh weight and amplitude *A* **(A)** and difference of the locking phase |Δ*θ*_1_| **(B)** in +10°C. **(C,D)** Relationship between fresh weight and *A* **(C)** and |Δ*θ*_1_| **(D)** in the −10°C. The blank symbols indicate data with negative values. Dotted arrows indicate the transform from negative to positive values. The colored symbols indicate data with positive values and the absolute value of the negatives in **(B,D)**. The solid lines indicate regression lines. **(B,D)** The regression lines are for the colored symbols.

In Figures 6A,B, the relationship between fresh weight and the *CCA1* rhythm in the +10°C conditions is shown. The fresh weight showed a linear relationship for the amplitude of *A* (Figure 6A) but a mirrored linear relationship for the locking phase *θ*_1_ (Figure 6B). Thus, we introduced the absolute value of phase difference |Δ*θ*_1_| defined as $\left|\Delta \,\theta_{1}\right|=\left|\theta_{1}-{\bar{\theta }}_{1,C}\right|$, which indicates the amount of phase shift forced by a +10°C cycle (Figure 6B).$\,{\bar{\theta }}_{1,C}$ is the average of *θ*_1_ under the control conditions. Notably, the fresh weight showed very high correlations with both *A* and |Δ*θ*_1_|. However, under the −10°C condition, the fresh weight showed no correlations with either *A* or |Δ*θ*_1_| (Figures 6C,D).

**FIGURE 7:**
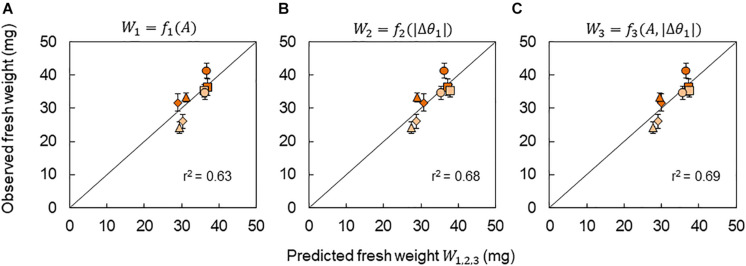
Prediction of fresh weight based on the *CCA1* rhythms. Correlation between observed fresh weight and predicted fresh weight using models *W*_1_ = *f*_1_(*A*) **(A)**, *W*_2_ = *f*_2_(|Δ*θ*_1_|) **(B)**, and *W*_3_ = *f*_3_(*A*, |Δ*θ*_1_|) **(C)**. The value of *r*^2^ indicates the coefficient of determination for the line with a slope of 1. Symbols indicate the same conditions as shown in Figure 6.

Due to the strong correlation between the growth and the*CCA1* rhythm (*A* and |Δ*θ*_1_|) under the +10°C condition, a growth prediction based on the *CCA1* rhythm could be demonstrated. In this study, three kinds of prediction model for fresh weight were considered: *W*_1_ = *f*_1_(*A*), *W*_2_ = *f*_2_(|Δ*θ*_1_|), and *W*_3_ = *f*_3_(*A*, |Δ*θ*_1_|), where *W*_1,2,3_ means the predicted fresh weight in each model. First, the functions *f*_1_(*A*) and *f*_2_(|Δ*θ*_1_|) were obtained experimentally as linear functions from the relationships in Figures 6A,B, respectively. The function *f*_3_(*A*, |Δ*θ*_1_|) was also obtained by a linear optimal combination of *A* and |Δ*θ*_1_|, that is, *f*_3_(*A*, |Δ*θ*_1_|) = *aA* + *b*|Δ*θ*_1_| + *c*, using the least square method. We then calculated the *A* and |Δ*θ*_1_| for each Δ*t* in the phase oscillator model (Eq. 7) and the regression lines in Figures 3A,B as a calibration. Finally, *A*(Δ*t*) and |Δ*θ*_1_(Δ*t*)| were obtained and substituted into *W*_1_ = *f*_1_[*A*(Δ*t*)], *W*_2_ = *f*_2_[|Δ*θ*_1_(Δ*t*)|], or *W*_3_ = *f*_3_[*A*(Δ*t*), |Δ*θ*_1_(Δ*t*)|], and then we obtained the predicted fresh weight for each Δ*t*. The accuracy rates of the models were high (*r* > 0.79) as shown in Figure 7. However, it should be noted that our prediction model cannot be used for the −10°C condition, as there was no correlation between growth and the *CCA1* rhythm.

## Discussion

In this study, we investigated the modulation of the *CCA1* rhythms and seedling growth using temperature cycles (4 h ± 10°C) with time lags Δ*t*. The amplitude *A* and locking phase *θ*_1_ varied, depending on Δ*t*. Under the −10°C condition, there was a decrease in the modulation of *A* and *θ*_1_ by 53 and 75%, respectively, compared with those under the +10°C condition (Figure 2). In addition, the growth depended on Δ*t* only under the +10°C condition, and not under −10°C (Figure 4). Notably, under the +10°C condition, growth showed a high correlation with the *CCA1* rhythms (Figure 6).

**FIGURE 8:**
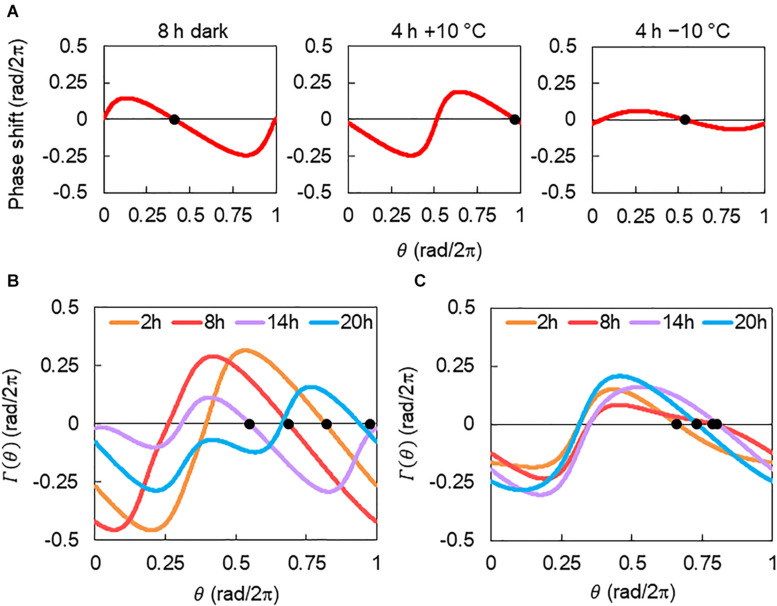
Description of combined phase response curve (PRC). **(A)** PRCs for 8 h dark and 4 h ± 10°C stimuli. PRCs were reconstructed with *a* = 0.20 and *α* = 0.13 for 8 h darkness, *a* = 0.39 and *α* = 0.58 for 4 h +10°C, and *a* = 0.10 and *α* = 0.13 for 4 h −10°C stimuli (Masuda et al., 2021). Black dots indicate the stable points. **(B)** The combined PRC Γ(*θ*) of 8 h dark and 4 h +10°C condition. **(C)** Γ(*θ*) of 8 h dark and 4 h −10°C condition. Each curve corresponds to Γ(*θ*) for Δ*t* = 2, 8, 14, or 20 h. Black dots indicate the stable point of each Γ(*θ*).

In our model, light and temperature stimuli affected the *CCA1* rhythms via their PRCs. Consequently, their multiple effects can be estimated based on the combined PRC Γ(*θ*). Figure 8A shows the description of Γ(*θ*). PRCs for 8 h dark, 4 h +10°C, and 4 h −10°C were obtained in our pervious study (Masuda et al., 2021). Here, we introduced the combined PRC Γ(*θ*), described as Γ(*θ*) = *G*_D_(*θ* + *θ_D_*) + *G*_T_(*θ* + Δ*ψ*), where *G*_D_(*θ*) and *G*_T_(*θ*) are the PRCs for the 8 h dark and temperature (4 h +10°C or 4 h −10°C) stimuli, respectively. *θ_D_* is a constant that denotes the start time of the dark in relation to the timing of light on, that is, $\theta_{\text{D}}=2\,\pi^{*}\,\frac{16}{24}$ (rad) in this study. Δ*ψ* is the phase delay of the temperature stimuli from light on; Δ*ψ* = 0 rad indicates the temperature stimulus applied at the light-on time. Δ*ψ* was transferred to time lag Δ*t* as Δ*t* = Δ*ψ*/2*π* × 24 (h) in this study. By considering the Γ(*θ*), the differences in rhythm modulations between +10°C and −10°C were determined and shown in Figure 3C. They were explained as follows: the shape of Γ(*θ*) changes drastically depending on Δ*t* in +10°C (Figure 8B), but it was almost unchanged at −10°C (Figure 8C). Therefore, in the −10°C condition, the *CCA1* rhythm was determined to be almost independent of the time lag. In addition, the complex shape of Γ(*θ*), such as Δ*t* = 14 and 20 h under +10°C, disturbs the synchrony of cellular oscillators and then suppresses substantially the amplitude of the collective rhythm (around evening in Supplementary Figure S2A).

During plant growth, Δ*t* also resulted in a significant effect at the +10°C but not −10°C condition (Figure 4). In addition, the growth showed a correlation with the *CCA1* rhythms at +10°C, but no correlation at −10°C (Figure 6). As a possible explanation, the variable range for the −10°C was very narrow (on a small loop in Figure 3C), so the growth might not be affected by the rhythm modulations. Moreover, using Δ*t*, the degree of DIF was regulated continuously from +DIF to −DIF. Such continuous regulation of DIF provides continuous modulation of the *CCA1* rhythm (Figure 3C). Based on our results, the effects of DIF regulation on growth were predictable (Figure 7). This prediction method in conjunction with specific growing conditions and/or crop species might be important for practical application in horticulture (Kläring and Schmidt, 2017; Liu et al., 2019). However, as a limitation of this, other situations such as the 12/12 h temperature cycles were not addressed in this study, and further studies are required to determine the growth regulation of DIF.

In this study, we demonstrated growth prediction using the *CCA1* rhythm with a phase oscillator model. The synchronization of the cellular oscillators plays an important role in the modification of circadian rhythms. Although it is difficult to measure such population dynamics directly, our model was utilized for the basic estimation of the optimal time lag. However, although the present study showed a correlation between growth and the *CCA1* rhythms, it did not clarify a causal mechanism between them. The measurement of other clock genes is required to fully elucidate the role of the circadian clock. Moreover, the entrainment of the plant circadian clock also involves complex interactions for endogenous factors (e.g., sugar metabolism and hormone signaling) as well as environmental stimulus (Webb et al., 2019). Further modeling with signaling details and other environmental stimuli is thus required in the future.

## Data Availability Statement

The raw data supporting the conclusions of this article will be made available by the authors, without undue reservation.

## Author Contributions

KM, TY, and HF designed the research. KM, TY, and YK performed the experiments. KM and TY analyzed the data. KM and HF wrote the manuscript. All authors discussed the results and implications and commented on the manuscript.

## Conflict of Interest

The authors declare that the research was conducted in the absence of any commercial or financial relationships that could be construed as a potential conflict of interest.
