# Supplementary material for: Time Lag Between Light and Heat Diurnal Cycles Modulates CIRCADIAN CLOCK ASSOCIATION 1 Rhythm and Growth in Arabidopsis thaliana
Source: Front Plant Sci. 2021 Feb 11;11:614360. doi: 10.3389/fpls.2020.614360 (PMC7905214; doi:10.3389/fpls.2020.614360)
Supplement: Supplementary file 1 [file Data_Sheet_1.PDF]

# Supplementary Material

## 1 Supplementary Figures

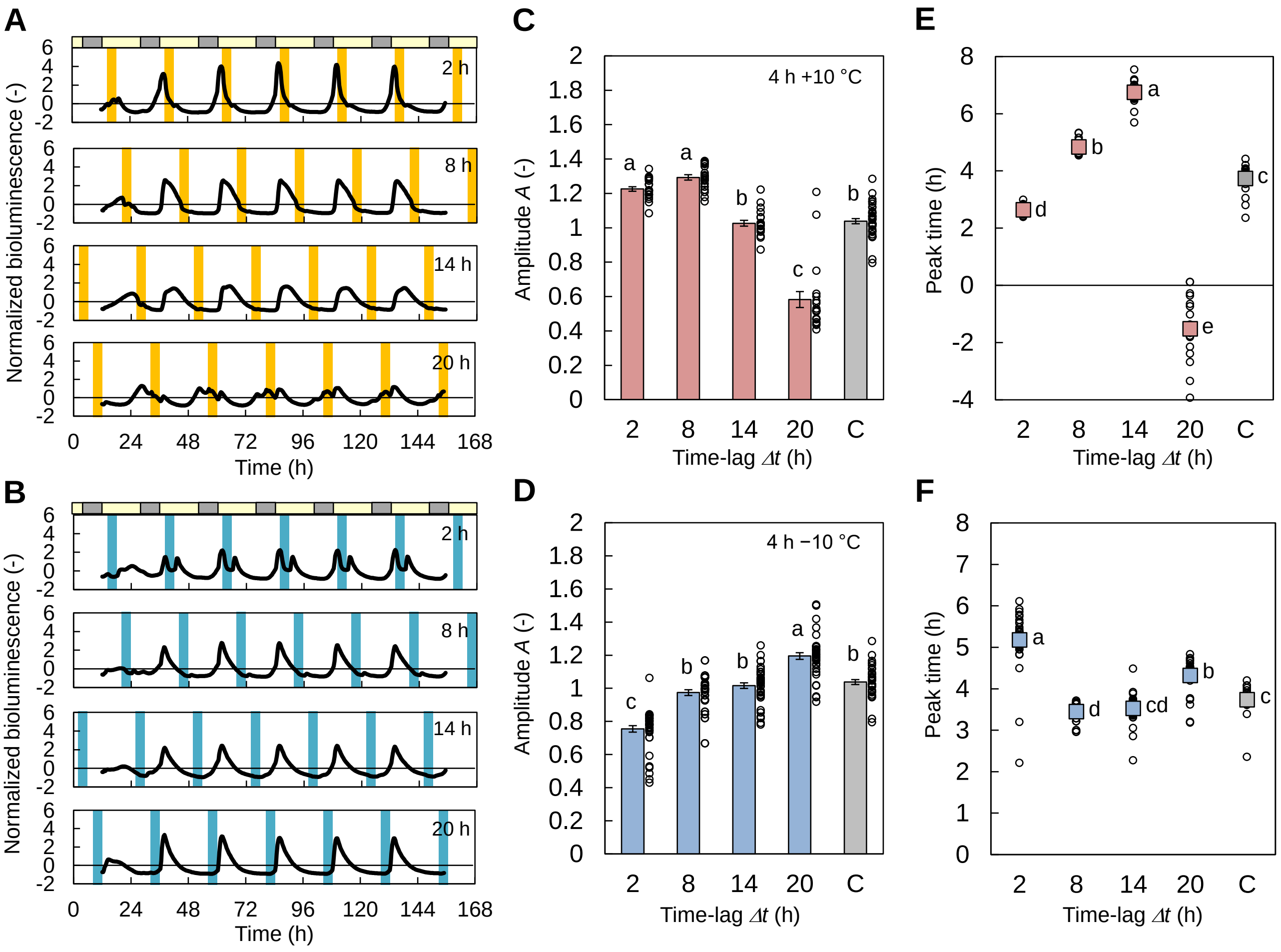


**Figure S1.** Effects of the time lag on *CCA1* rhythms under conditions of light-dark (L/D) = 16/8 h and 4 h ±10 °C cycles with anti-phase initial conditions. **(A, B)** Normalized bioluminescence of *CCA1*::*LUC* under +10 °C **(A)** and −10 °C **(B)** conditions. **(C, D)** Amplitude *A* of the bioluminescence oscillation under +10 °C **(C)** and −10 °C **(D)** conditions (mean ± SEM, $n = 20$ individuals in +10 °C, 40 in −10 °C, and 40 in the control). The control is labelled C. The circles indicate the individual data points. Different letters indicate significant differences for each panel (Tukey-Kramer test, $p < 0.05$). **(E, F)** Peak time under +10 °C **(E)** and −10 °C **(F)** conditions (circular mean, $n = 20$ individuals in +10 °C, 40 in −10 °C, and 40 in the control). The circles indicate the individual data points. Different letters indicate significant differences for each panel (Watson-Williams test with Bonferroni correction, $p < 0.05$).

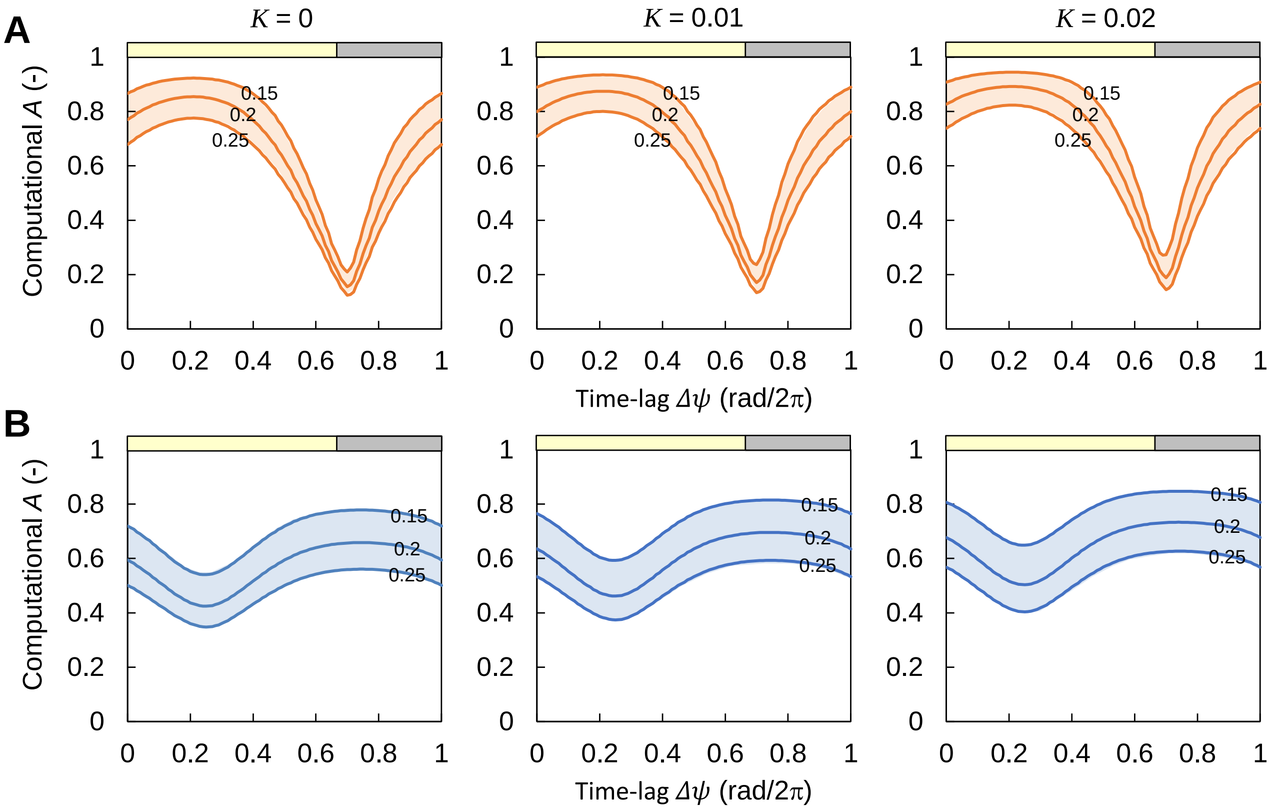


**Figure S2.** Computational amplitudes for different values of the coupling strength and distributions of the circadian frequency. **(A)** Computational amplitude for each time lag at $K$ = 0.00, 0.01, and 0.02 under +10 °C conditions. Each line indicates amplitude at $\sigma_\omega/\omega_0$ = 0.15, 0.20, or 0.25. **(B)** Computational amplitude for each time lag at $K$ = 0.00, 0.01, and 0.02 under −10 °C conditions. Each line indicates an amplitude at $\sigma_\omega/\omega_0$ = 0.15, 0.20, or 0.25.
